# Headache service quality: evaluation of quality indicators in 14 specialist-care centres

**DOI:** 10.1186/s10194-016-0707-9

**Published:** 2016-12-08

**Authors:** Sara Schramm, Derya Uluduz, Raquel Gil Gouveia, Rigmor Jensen, Aksel Siva, Ugur Uygunoglu, Giorgadze Gvantsa, Maka Mania, Mark Braschinsky, Elena Filatova, Nina Latysheva, Vera Osipova, Kirill Skorobogatykh, Julia Azimova, Andreas Straube, Ozan Emre Eren, Paolo Martelletti, Valerio De Angelis, Andrea Negro, Mattias Linde, Knut Hagen, Aleksandra Radojicic, Jasna Zidverc-Trajkovic, Ana Podgorac, Koen Paemeleire, Annelien De Pue, Christian Lampl, Timothy J. Steiner, Zaza Katsarava

**Affiliations:** 1 Institute for Medical Informatics, Biometry and Epidemiology, University Hospital of Duisburg-Essen, Hufelandstr 55, 45122 Essen, Germany; 2 Department of Neurology, Cerrahpasa School of Medicine, Istanbul University, Istanbul, Turkey; 3 Hospital da Luz Headache Centre, Neurology Department, Hospital da Luz, Lisbon, Portugal; 4 Danish Headache Centre, Department of Neurology, University of Copenhagen, Rigshospitalet-Glostrup, Copenhagen, Denmark; 5 Department of Neurology, Aversi Clinic, Tbilisi, Georgia; 6 Department of Neurology, Tartu University Clinics, Tartu, Estonia; 7 Alexander Vein Headache Clinic, Moscow, Russian Federation; 8 I.M. Sechenov First Moscow State Medical University, Moscow, Russian Federation; 9 University Headache Clinic, Moscow, Russian Federation; 10 Department of Neurology, Ludwig-Maximilians-University Munich, Klinikum Großhadern, Munich, Germany; 11 Department of Clinical and Molecular Medicine, Sapienza University of Rome, Regional Referral Headache Centre, Sant’Andrea Hospital, Rome, Italy; 12 Department of Neuroscience, Norwegian University of Science and Technology, Trondheim, Norway; 13 Norwegian Advisory Unit on Headaches, St Olavs University Hospital, Trondheim, Norway; 14 Neurology Clinic Clinical Centre of Serbia, Belgrade, Serbia; 15 Faculty of Medicine, University of Belgrade, Belgrade, Serbia; 16 Department of Neurology, Ghent University Hospital, Ghent, Belgium; 17 Headache Medical Center Linz Seilerstätte, Linz, Austria; 18 Division of Brain Sciences, Imperial College London, London, UK; 19 Department of Neurology, Evangelical Hospital Unna, Unna, Germany; 20 Medical Faculty, University of Duisburg-Essen, Essen, Germany

**Keywords:** Headache disorders, Headache care, Service quality evaluation, Global campaign against headache

## Abstract

**Background:**

The study was a collaboration between *Lifting The Burden* (LTB) and the *European Headache Federation* (EHF). Its aim was to evaluate the implementation of quality indicators for headache care Europe-wide in specialist headache centres (level-3 according to the EHF/LTB standard).

**Methods:**

Employing previously-developed instruments in 14 such centres, we made enquiries, in each, of health-care providers (doctors, nurses, psychologists, physiotherapists) and 50 patients, and analysed the medical records of 50 other patients. Enquiries were in 9 domains: diagnostic accuracy, individualized management, referral pathways, patient’s education and reassurance, convenience and comfort, patient’s satisfaction, equity and efficiency of the headache care, outcome assessment and safety.

**Results:**

Our study showed that highly experienced headache centres treated their patients in general very well. The centres were content with their work and their patients were content with their treatment. Including disability and quality-of-life evaluations in clinical assessments, and protocols regarding safety, proved problematic: better standards for these are needed. Some centres had problems with follow-up: many specialised centres operated in one-touch systems, without possibility of controlling long-term management or the success of treatments dependent on this.

**Conclusions:**

This first Europe-wide quality study showed that the quality indicators were workable in specialist care. They demonstrated common trends, producing evidence of what is majority practice. They also uncovered deficits that might be remedied in order to improve quality. They offer the means of setting benchmarks against which service quality may be judged. The next step is to take the evaluation process into non-specialist care (EHF/LTB levels 1 and 2).

**Electronic supplementary material:**

The online version of this article (doi:10.1186/s10194-016-0707-9) contains supplementary material, which is available to authorized users.

## Background

Headache disorders are a major public-health problem. They are common, give rise to widespread ill-health [18] and are the third highest cause of disability worldwide [16, 20]. Through lost productivity, they impose substantial financial burdens on individuals and society: in Europe their annual cost exceeds EUR 100 billion [5]. They are largely treatable, but effective care fails to reach most people worldwide [16, 22].

Although the scope, character and content of headache practice have changed markedly during recent years, the principal driver has been the introduction of new drugs. While the need for better education of medical professionals has been authoritatively proclaimed as a prerequisite and high priority for improving headache care [22], adequacy of care in its broadest sense has not tended to engage social awareness. This must change: the concept of service quality needs to take centre-stage in headache care, and become the driver for both headache centres and health-care providers (HCPs) in primary care, who manage most patients with headache [14].

This study continues the service quality evaluation collaborative project between European Headache Federation (EHF) and *Lifting The Burden* (LTB), within the Global Campaign against Headache [10, 11], which is conducted by LTB in official relations with the World Health Organization (WHO).

Quality assessment of headache care requires contributions from three parties: HCPs, other health-care workers and patients. As a collaborative group of health-services researchers and headache specialists, we first defined quality in the context of headache care [7], then formulated a set of quality indicators based on this definition that might be applied to headache care across countries, cultures and settings [8]. The ultimate purpose was that inadequacies in headache care worldwide might be recognized and rectified. Taking account of the attributes of care and of criteria for what constitutes good care, we proposed a multidimensional definition of quality:

“Good quality headache care achieves accurate diagnosis and individualized management, has appropriate referral pathways, educates patients about their headaches and their management, is convenient and comfortable, satisfies patients, is efficient and equitable, assesses outcomes and is safe” [7].

In a pilot study in two specialist headache centres in Germany and Portugal, we evaluated quality indicators, and related assessment instruments, in nine domains aligning with this definition (see Table 1) [3]. We interrogated HCPs and patients, finding that the enquiry was feasible and the questionnaires were easily understood by both groups, without being unduly time-consuming. The results from the two centres were intuitively meaningful, and comparable despite their very different settings (one university hospital-based, the other private hospital-based) [3].

**Table 1:** The nine domains of quality in a headache service (from reference [8])

Domain A:	Diagnostic accuracy, therefore asking whether diagnosis were made according to the IHS criteria, documented during the first visit and reviewed during the follow-ups and supported by the diagnostic diaries.
Domain B:	Issues of the individualized management including waiting time, use of diaries and instruments of headache related disability in treatment plans.
Domain C:	Availability and utilization of urgent and specialist referral pathways.
Domain D:	Patient’s education and reassurance
Domain E:	Convenience, comfort and welcoming of the clinic
Domain F:	Patient’s satisfaction
Domain G:	Equity and efficiency of the headache care including access to care, wastage of resources, rate of technical investigations and costs.
Domain H:	Outcome measures including clinical parameters but also measures of disability and quality of life.
Domain I:	Safety of care

*IHS* International Headache Society

The present study extends this evaluation Europe-wide, and involves a range of competencies between leading academic specialist headache centres with advanced multidisciplinary care delivered by headache specialists (level-3 according to the EHF/LTB standard [14]) and non academic centres with ambulatory care delivered by physicians with a special interest in headache (level-2 [14]). The aims were to demonstrate wider acceptability of the instruments and feasibility of use across countries and settings, and their fitness for purpose and utility as headache service quality indicators.

## Methods

### Ethics approval

Each participating centre applied for ethics approval according to local regulations. Since the primary purpose of the study was service quality improvement, in some countries it was deemed to fall outside the scope of research ethics review. Informed consent was obtained from all study participants (service staff and interviewed patients) regardless of whether or not ethics approval had been required. Data relating to individual patients were collected anonymously and held in accordance with European data-protection legislation.

### Study settings and participating centres

We sent invitations to 23 secondary (level-2) or tertiary (level-3) headache clinics in 14 countries, selecting centres known to us as providing expert care and likely to be able to participate. One German clinic declined (not wishing to spend time applying for ethics approval), as did another in Germany, one in the USA, one in Serbia and six in the Netherlands for unknown reasons. Fourteen clinics in 12 countries of Europe participated over 8 months between February and September 2014. They are described in Table 2.

**Table 2:** Characteristics of headache centres

No	Country	City	Centre name	Description	Level^a^
1	Austria	Linz	Headache Medical Center Linz Seilerstätte	Headache service run by one headache-experienced neurologist with link to the department of neurogeriatric medicine in the Hospital Barmherzige Schwester Linz, link to multidisciplinary approach (headache nurse, psychologist and physiotherapist) within the hospital.	3
2	Belgium	Ghent	Ghent University Hospital, Department of Neurology	Training hospital-based headache service run within the neurology department by one headache-experienced neurologist; most patients are seen by supervised residents (of whom there are up to 6). No multidisciplinary approach: the service has a study nurse but no psychologist or physiotherapist.	3
3	Denmark	Copenhagen	Danish Headache Centre, University of Copenhagen, Glostrup	Academic tertiary headache centre in a university hospital, and a national referral centre for patients with refractory or rare headache disorders and cranial neuralgias. Staffed by 7 headache-experienced neurologists, 3 psychologists, 3 physiotherapists and 4 study nurses.	2 and 3
4	Estonia	Tartu	Tartu University Clinics, Department of Neurology, Headache Clinic	Academic university-based clinic run by two physicians and two headache nurses. Options within the hospital for multidisciplinary care avaliable with limitations: physiotherapy must be paid for by patients (not covered by national insurance); specialist psychology (again to be paid for) is available from another clinic of the institution.	3
5	Georgia	Tbilisi	Aversi Clinic	Operated within the private sector as a stand-alone headache centre by 3 headache-experienced neurologists supported by a psychologist, physiotherapist and study nurse.	2
6	Germany	Munich	Upper Bavarian Headache Center, Hospital of the Ludwig-Maximilians-University (LMU) Munich, Campus Großhadern	Hospital-based headache service provided by 3 headache-experienced physicians supported by one psychologist, 3 physiotherapists and a study nurse.	3
7		Unna	Evangelisches Krankenhaus Unna, Department of Neurology	Hospital-based headache clinic run within the department of neurology by one headache-experienced neurologist supported by 3 psychologists, physiotherapists and a study nurse.	2
8	Italy	Rome	Regional Referral Headache Centre, Sant’Andrea Hospital	Hospital-based headache service run by 4 headache-experienced physicians (2 internists, one rheumatologist, one psychiatrist) supported by one post-graduate internist trainee, 3 psychologists and 2 nurses.	3
9	Norway	Trondheim	Department of Neuroscience, Norwegian University of Science and Technology; Norwegian Advisory Unit on Headache, St Olavs University Hospital	University hospital-based service and national advisory centre run by two headache-experienced neurologists with support from a specialist nurse. Hospital provides all options for multidisciplinary care.	3
10	Portugal	Lisbon	Hospital Da Luz Headache Center	Private hospital-based service run by 2 headache-experienced neurologists. Hospital is not departmentalized: many specialties share space; 6 gynaecologists, a dentist, a maxillofacial surgeon, 2 physiatrists and 2 psychiatrists have special interests in headache, offering fast referral. Four physical therapists, a psychologist and a nurse also see headache patients.	3
11	Russian Federation	Moscow	Alexander Vein Headache Clinic	Private headache clinic employing 9 neurologists experienced in headache medicine, supported by 4 psychiatrists, 3 manual therapists, a biofeedback specialist and acupuncture specialists. The Clinic also runs a Botox headache service.	2 and 3
12	Russian Federation	Moscow	University Headache Clinic	Private university-based headache service and training centre run by 5 headache-experienced neurologists supported by 2 psychiatrists and 2 physiotherapists and nurses.	3
13	Serbia	Belgrade	Neurology Clinic Clinical Centre of Serbia, Faculty of Medicine, University of Belgrade	University-based headache service run by 2 headache specialists with part-time engagement, without the support of a trained nurse.	3
14	Turkey	Istanbul	Istanbul University, Department of Neurology	University-based headache service and training centre run by 5 headache experts supported by a psychologist, study nurse and nurse for assisting in interventional treatments.	3

*EHF* European headache federation, *LTB* lifting the burden

^a^According to the EHF/LTB standard [14]

### Study participants

The subject of study at each centre was the quality of service. Service staff (doctors [including trainees, where appropriate], other HCPs [nurses, psychologists, physiotherapists, others], the service manager [the person responsible for ensuring the service was properly run and maintained, who might be the clinical chief or a health-service manager] and the appointments administrator [usually a nurse or secretary]) and patients were witnesses; they were therefore the study participants, although not themselves objects of study.

The patient participants at each centre were a prospective consecutive sample (*n* = 50). In addition, information was acquired from the records of a retrospective random or consecutive sample of 50 patients other than those seen prospectively.

### Study instruments

There were five questionnaires: one each for doctors, other HCPs, the service manager, the appointments administrator and patients. The last took the form of an exit questionnaire. This and the questionnaire for other HCPs (when necessary) were translated into the local language(s) by the local investigators according to LTB’s translation protocols [6]. Questionnaires are attached in Additional files 1, 2, 3, 4 and 5. In addition, some items of information (see Table 3) were extracted from patients’ or service records in a retrospective review by a staff member of the service using a sixth instrument. Table 3 shows the methods of implementation of quality indicators.

**Table 3:** Methods of implementation of quality indicators

	Indicator	Measure	Application
Domain A. Accurate diagnosis is essential for optimal headache care			
A1	Patients are asked about the temporal profile of their headaches	a) Duration of presenting complaint is recorded in patient’s record (yes/no) b) Frequency or days/month of symptoms is recorded in patient’s record (yes/no)	Review of relevant fields in records of retrospective (random or consecutive) sample of patients (*n* = 50)
A2	Diagnosis is according to current ICHD criteria	a) Diagnosis is recorded in patient’s record (yes/no) b) Diagnostic record uses ICHD terminology (yes/no)	
A3	A working diagnosis is made at the first visit	Working diagnosis at first visit is recorded in patient’s record (yes/no)	
A4	A definitive diagnosis is made at first or subsequent visit	Definitive diagnosis is recorded in patient’s record or, if not, an appointment for review has been given (yes/no)	
A5	Diagnosis is reviewed during later follow-up	Diagnostic review during follow-up is routinely undertaken (yes/no)	Enquiry of doctors
A6	Diaries are used to support or confirm diagnosis	The service has a diagnostic diary available and doctors are aware of its availability (yes/no)	Enquiry of service manager and doctors into availability
Domain B. Individualized management is essential for optimal headache care			
B1	Waiting-list times for appointments are related to urgency of need	a) A formal triage system exists (yes/no) b) To expedite appointments in cases of perceived urgency (yes/no)	Enquiry of doctors, service manager and appointments administrator
B2	Sufficient time is allocated to each visit for the purpose of good management	a) Actual time (minutes) per visit is recorded by patient in exit questionnaire b) Satisfaction (yes/no) with actual time is recorded by patient in exit questionnaire c) HCPs express overall satisfaction (yes/no)	a/b) Review of questionnaires from prospective consecutive sample of patients (*n* = 50) c) Enquiry of HCPs
B4	Treatment plans include psychological approaches to therapy when appropriate	Access route to psychological therapies exists and doctors are aware of its availability (yes/no/not applicable)	Enquiry of service manager and doctors into availability
B5	Treatment plans reflect disability assessment	An instrument for disability assessment is available and HCPs are aware of its availability (yes/no)	Enquiry of service manager and doctors into availability
B6	Patients are followed up to ascertain optimal outcome	a) The service permits follow-up as needed (yes/no) b) A follow-up diary and/or calendar is available (yes/no)	Enquiry of service manager and HCPs
Domain C. Appropriate referral pathways are essential for optimal headache care			
C1	Referral pathway is available from primary to specialist care	A usable pathway exists and doctors and appointments administrator are aware of its existence (yes/no)	Enquiry of service manager, doctors and appointments administrator into availability
C2	Urgent referral pathway is available when necessary	A usable pathway exists and doctors and appointments administrator are aware of its existence (yes/no)	Enquiry of service manager, doctors and appointments administrator into availability
Domain D. Education of patients about their headaches and their management is essential for optimal headache care			
D1	Patients are given the information they need to understand their headache and its management	a) Information leaflets are available (yes/no) and doctors and appointments administrator are aware of their existence (yes/no) b) Doctors provide patients with information (yes/no) c) Information was understandable (yes/no) d) Amount of information was about right (yes/no)	a) Enquiry of service manager, doctors and appointments administrator into availability b) Review of questionnaires from prospective consecutive sample of patients (*n* = 50) c/d) Review of questionnaires from prospective consecutive sample of patients (*n* = 50)
D2	Patients are given appropriate reassurance	Satisfaction (yes/no) with reassurance given is recorded by patient in exit questionnaire	Review of questionnaires from prospective consecutive sample of patients (*n* = 50)
Domain E. Convenience and comfort are part of optimal headache care			
E1	The service environment is clean and comfortable	a) Satisfaction (yes/no) with cleanliness and comfort is recorded by patient in exit questionnaire b) HCPs are satisfied with cleanliness and comfort (yes/no)	a) Review of questionnaires from prospective consecutive sample of patients (*n* = 50) b) Enquiry of HCPs
E2	The service is welcoming	Satisfaction (yes/no) with welcome is recorded by patient in exit questionnaire	Review of questionnaires from prospective consecutive sample of patients (*n* = 50)
E3	Waiting times in the clinic are acceptable	a) Actual waiting time (minutes) per visit is recorded by patient in exit questionnaire b) Satisfaction (yes/no) with waiting time is recorded by patient in exit questionnaire c) HCPs are satisfied with waiting times (yes/no)	a/b) Review of questionnaires from prospective consecutive sample of patients (*n* = 50) c) Enquiry of HCPs
Domain F. Achieving patient satisfaction is part of optimal headache care			
F1	Patients are satisfied with their management	Satisfaction (yes/no) with overall management is recorded by patient in exit questionnaire	Review of questionnaires from prospective consecutive sample of patients (*n* = 50)
Domain G. Optimal headache care is efficient and equitable			
G1	Procedures are followed to ensure resources are not wasted	A protocol to limit wastage exists (yes/no)	Enquiry of service manager
G2	Costs of the service are measured as part of a cost-effectiveness policy	A record of input costs exists (yes/no)	Enquiry of service manager
G3	There is equal access to headache services for all who need it	A policy to ensure equal access exists (yes/no)	Enquiry of service manager and HCPs
Domain H. Outcome assessment is essential in optimal headache care			
H1	Outcome measures are based on self-reported symptom burden (headache frequency, duration and intensity)	An outcome measure (HURT or similar) is available and HCPs are aware of its existence (yes/no)	Enquiry of service manager and HCPs
H2	Outcome measures are based on self-reported disability burden	An outcome measure (HALT or similar) is available and HCPs are aware of its existence (yes/no)	
H3	Outcome measures are based on self-reported quality of life	An outcome measure (WHOQoL or similar) is available and HCPs are aware of its existence (yes/no)	
Domain I. Optimal headache care is safe			
I1	Systems are in place to be aware of serious adverse events^a^	A system or protocol exists and HCP are aware of its existence (yes/no)	Enquiry of service manager and HCPs

*HCPs* health-care providers, *ICHD* international classification of headache disorders, *HURT* headache under-response to treatment questionnaire [1, 21], *HALT* headache-attributed lost time questionnaire [13], *WHOQoL* the world health organization quality of life questionnaire [9]

^a^Serious adverse events are those that cause death, are life-threatening, terminate or put at risk a pregnancy, or cause hospitalization, prolonged illness, disability and/or malignancy

### Procedure

At each centre, all staff completed the relevant survey questionnaires, doctors and other HCPs responding anonymously. Patients received their questionnaires (with age, gender and principal diagnosis pre-recorded) from nursing or administrative staff as they exited their consultations. They were encouraged to communicate their experiences and points of view in the questionnaire, completing it anonymously and returning it before leaving. Data extraction from the charts, records or electronic database of the retrospective sample was performed by a doctor, nurse or research assistant, under the supervision of the local principal investigator, using the study instrument provided for this purpose.

### Data management and analysis

Data were entered locally in each centre into spreadsheets provided, and the completed spreadsheets transferred to the central data collection centre (Institute for Medical Informatics, Biometry and Epidemiology, University Hospital of University Duisburg-Essen) where they were merged and analyzed by Sara Schramm. Demographic and clinical data were provided as numerical values and summarised as percentages or mean values with standard deviations (SDs). Analyses were descriptive only, with no statistical tests performed. Analyses were completed in Microsoft EXCEL 2010.

### Highlighted problems and feedback from centres

After data analysis we sent the findings, highlighting any problems indicated by them, to all participating centres for explanation or other feedback. Specifically, each centre was asked for a short commentary on these problems and to explain apparent quality deficits (citing, where apropriate, mitigating factors or obstacles to good-quality care built into the local health-care environment). These responses are presented with commentary.

## Results

Table 4 shows the characteristics of the 14 study centres and their participating patients. The date of completion of all items was between 3 February 2014 and 17 November 2014 with one exception: the records review of Russia-Moscow University was initially missing, and completed later between 2 and 5 October 2015. With different structures, the centres all had one manager but between one and 7 doctors, 0 and 16 nurses and other HCPs and one and 8 secretarial or administrative staff. Centres returned questionnaires from 30 to 58 patients, with mean age ranging from 34.9 years in Russia-Moscow University to 46.3 years in Denmark-Copenhagen. Mean duration of the presenting headache disorder ranged from 6.8 years in Georgia-Tbilisi to 17.6 years in Norway-Trondheim. The spectrum of diagnoses was very different across centres (Table 4). Each centre analyzed between 34 and 58 retrospective records.

**Table 4:** Characteristics of participating centres and patients

	Headache centre	Austria Linz	Belgium Ghent	Denmark Copenhagen	Estonia Tartu	Georgia Tbilisi	Germany Munich	Germany Unna	Italy Rome	Norway Trondheim	Portugal Lisbon	Russia Moscow AV	Russia Moscow U	Serbia Belgrade	Turkey Istanbul
Staff	Manager, n	1	1	1	1	1	1	1	1	1	1	1	1	1	1
	Administative staff, n	1	3	1	1	2	2	1	1	0	8	2	3	4	1
	Doctors, n	1	7	4	2	2	3	1	2	2	4	3	5	3	2
	Other HCPs, n	1	0	16	2	2	4	2	3	1	3	1	4	0	2
Patients	Patients, n	58	50	51	50	50	53	50	51	30	52	50	50	50	50
	Mean age (years) ± SD	38.8 ± 16.6	40.2 ± 15,3	46.3 ± 15.6	39.8 ± 14.7	35.5 ± 13.2	40.6 ± 15.4	41.2 ± 14.9	42.8 ± 13.9	42.8 ± 14.4	36.7 ± 10.9	42.8 ± 12.9	34.9 ± 10.5	44.4 ± 13.4	38.8 ± 10.2
	Mean duration of headache, years ± SD	13.4 ± 11.7	10.5 ± 12.1	12.6 ± 14.4	11.3 ± 11.1	6.8 ± 6.3	15.6 ± 14.1	12.7 ± 14.2	16.3 ± 12.6	17.6 ± 16.9	17.5 ± 13.2	13.3 ± 12.6	11.3 ± 9.3	11.6 ± 11.7	11.6 ± 7.7
	Records reviewed, n	58	50	50	50	50	50	49	51	34	51	50	50	50	50
	Diagnoses, n														
	Migraine	36	36	17	26	34	44	39	36	27	41	30	41	22	38
	TTH	3	1	7	14	4	0	2	4	0	6	14	4	12	5
	Trigeminal neuralgia	4	1	3	0	2	0	1	0	0	0	0	0	2	4
	Cluster headache	2	3	7	0	2	1	2	0	2	1	3	0	4	1
	MOH	3	0	6	1	0	0	0	0	0	0	0	0	6	0
	Other	10	8	8	8	8	0	5	0	1	4	3	5	4	2
	Missing	0	1	2	1	0	3	1	11	0	0	0	0	0	0

*HCPs* health care providers, *SD* standard deviation, *TTH* tension type headache, *MOH* medication-overuse headache, *AV* Alexander Vein, *U* University

On a practical level, the questionnaires in 10 different languages were reported as easy to apply and were understood and accepted by both HCPs and patients. They were not unduly time consuming. None of the specific enquiries caused or led to difficulties. Evaluation of each clinic according to the quality indicators is shown in Table 5. Findings are summarised below in domains. Problems highlighted at the centres and explanatory commentaries of each centre are listed in Additional file 6.

**Table 5:** Results of the questionnaires (% of positive answers)

Headache centre	Austria Linz	Belgium Ghent	Denmark Copenhagen	Estonia Tartu	Georgia Tbilisi	Germany Munich	Germany Unna	Italy Rome	Norway Trondheim	Portugal Lisbon	Russia Moscow AV	Russia Moscow U	Serbia Belgrade	Turkey Istanbul
A1a. Duration of complaint recorded	100	88	100	100	72	78	98	92	100	100	56	100	100	94
A1b. Frequency of symptoms recorded	100	100	94	100	78	76	100	100	100	100	68	100	100	96
A2a. Diagnosis recorded	100	90	100	100	92	98	100	100	100	100	96	100	100	98
A2b. ICHD terminology used	100	92	100	96	92	96	100	90	100	100	98	100	92	98
A3. Working diagnose at first visit recorded	100	98	94	100	64	98	100	100	100	100	98	100	100	80
A4. Definitive diagnosis or appointment for review	100	98	98	96	86	98	100	88	56	100	98	100	100	90
A5. Routinely diagnostic review during follow-up (doctors)	100	100	100	100	100	100	100	100	100	100	100	0	100	100
A6. Diagnostic diaries available (manager + doctors)	100	100	100	100	100	100	100	100	33	80	100	100	100	100
B1a. Formal triage system exists (manager + HCPs)	100	90	67	0	0	100	100	75	100	100	67	100	88	100
B1b. It expedites appointments of urgent cases (manager + HCPs)	100	100	80	50	0	100	100	100	100	92	67	56	100	100
B2a. Time per visit (minutes), mean ± SD	25.5 ± 13.5	23.0 ± 8.0	20.2 ± 2.2	15.0 ± 5.5	18.6 ± 9.5	30.0 ± 13.8	12.5 ± 5.0	21.0 ± 4.2	26.2 ± 12.0	22.6 ± 15.9	31.3 ± 14.2	28.0 ± 16.7	24.0 ± 5.2	15.0 ± 5.6
B2b. Satisfaction with time per visit (patients)	91	98	90	100	100	96	82	98	100	96	100	100	98	82
B2c. Satisfaction with time per visit (HCPs)	100	83	65	50	75	86	100	100	100	85	100	100	33	50
B4. Access route to psychological therapies exists (manager + doctors)	100	25	100	0	40	100	100	40	0	50	100	100	50	100
B5. Instrument for disability assessment available (manager + HCPs)	100	38	81	20	60	38	25	100	75	49	0	60	50	100
B6a. Follow-up service of every patient who needs it (manager + HCPs)	100	75	90	100	100	50	75	100	25	87	100	100	100	100
B6b. Follow-up diary/calender available (manager + HCPs)	100	100	100	100	100	100	75	100	100	79	100	100	100	100
C1. Referral pathway exists (manger + HCPs)	100	73	100	100	100	100	100	100	100	84	17	100	88	100
C2. Urgent referral pathway exists (manager + HCPs)	100	80	83	100	80	33	100	100	100	84	33	67	88	100
D1a. Information leaflets available (HCPs)	100	50	100	100	100	63	100	100	75	37	80	100	50	40
D1b. Doctor provides patient with information (patients)	100	100	90	96	100	100	94	100	97	100	100	100	98	98
D1c. Information given understandable (patients)	100	100	98	100	100	100	98	98	100	98	100	100	98	98
D1d. Amount of information about right (patients)	100	98	89	96	92	98	85	98	97	94	98	98	94	82
D2. Patients were given reassurance (patients)	100	98	59	90	100	89	100	98	100	100	100	98	98	96
E1a. Service environment clean and comfortable (HCPs)	100	100	20	0	100	100	100	100	100	100	75	100	67	100
E1b. Service environment clean and comfortable (patients)	100	98	84	98	100	92	96	100	100	100	100	100	92	80
E2. Satisfaction with welcome (patients)	98	98	100	98	100	96	100	100	100	98	100	100	98	100
E3a. Waiting time (minutes), mean ± SD	6.1 ± 1.2	17.0 ± 1.5	13.5 ± 1.3	13.3 ± 0.5	14.8 ± 0.2	12.6 ± 1.2	15.1 ± 1.2	18.2 ± 0.7	6.2 ± 1.3	15.6 ± 3.8	4.8 ± 0.7	4.4 ± 0.2	42.6 ± 5.4	33.1 ± 5.4
E3b. Satisfaction with waiting time (patients)	100	88	85	78	92	96	88	92	97	67	100	98	70	72
E3c. Satisfaction with waiting time (HCPs)	100	17	50	25	25	100	100	60	0	85	100	100	0	50
F1. Satisfaction with overall management (patients)	93	98	98	100	100	100	100	0,98	100	100	100	96	100	98
G1. Protocol to limit wastage exists (manager)	0	0	100	100	0	0	0	100	100	0	0	100	100	100
G2. Record of input costs exists (manager)	0	0	100	100	100	0	0	100	100	0	100	100	0	0
G3. Policy to ensure equal access exists (manager + HCPs)	100	63	67	80	60	25	100	50	50	50	40	0	75	100
H1. HURT or similar (manager + HCPs)	100	13	76	80	0	50	75	50	50	75	60	100	75	100
H2. HALT or similar (manager + HCPs)	100	25	70	80	0	57	0	50	50	75	40	70	75	40
H3. WHOQoL or similar (manager + HCPs)	100	0	76	80	40	57	0	50	0	63	20	100	25	0
I1. Protocol for reporting serious adverse events exists (manager + HCPs)	0	13	90	60	100	0	0	83	25	0	0	60	75	100

*HCPs* Health-care providers, *ICHD* International Classification of Headache Disorders, *HURT* Headache Under-Response to Treatment questionnaire [1, 21], *HALT* Headache-Attributed Lost Time questionnaire [13], *WHOQoL* World Health Organization Quality of Life questionnaire [9], *AV* Alexander Vein, *U* University

Domain A. Accurate diagnosis is essential for optimal headache care: The temporal profile of the headache was recorded in most clinics and diagnoses were made according to current International Classification of Headache Disorders (ICHD) criteria in more than 90% of cases in all headache centres. Not all centres had a system of working diagnosis at first visit, definitive diagnosis at first or subsequent visits, and review of the diagnosis during later follow up, which was explained by various organizational restrictions on opportunities to offer follow-up visits. Diagnostic diaries were available in all centres but Norway-Trondheim.

Domain B. Individualized management is essential for optimal headache care: Not all centres reported a formal triage system to expedite appointments in cases of perceived urgency, but this applied to disorders such as cluster headache rather than secondary headaches with serious underlying disorders. Mean time allocated to patients’ visits (according to patients’ reports) ranged between 12 and 31 min. Whereas the majority of the patients were satisfied with this allocated time, a high number of HCPs would have preferred more time per visit. In many centres there was no access route to psychological therapies and no instrument for disability assessment. In 4 centres, not all patients received follow-up who needed it because of the organizational barriers mentioned above. Follow-up diaries were available in almost all centres.

Domain C: Appropriate referral pathways are essential for optimal headache care: Our study identified a need for better referral pathways in some countries.

Domain D. Education of patients about their headaches and their management is essential for optimal headache care: Information leaflets were not available at all centres, although there were information leaflets in English freely available on the EHF-website [2] and leaflets in six European languages available from LTB [4]. Nevertheless, the great majority of patients expressed satisfaction with the information given by the doctor. Patients received appropriate reassurance in almost all cases.

Domain E. Convenience and comfort are part of optimal headache care: More patients than HCPs considered that the service environment was clean and comfortable; almost all patients felt welcomed. Waiting times in the clinics varied quite widely around means of 4–43 min, but were unsatisfactory for only a sizeable minority of patients. Regardless of this, up to 100% of HCPs were dissatisfied with the waiting time.

Domain F. Achieving patient satisfaction is part of optimal headache care: Overall satisfaction with their management was expressed by most patients, apparently regardless of waiting time and time allocated to their visits.

Domain G. Optimal headache care is efficient and equitable: Half of the clinics had protocols to avoid wastage of resources. Running costs were calculated in half of the clinics also. Only three clinics were able to offer equal access to headache services for all who might need it.

Domain H. Outcome assessment is essential in optimal headache care: Outcome assessment, also relevant to evaluation of cost-effectiveness in future health-care plans, required improvement in many of the centres. Quality-of-life measures in particular appeared to be underused.

Domain I. Optimal headache care is safe: Formal protocols to ensure reporting of serious adverse events were lacking in many centres.

## Discussion

This study was not planned primarily to assess service quality in the 14 centres but to evaluate the quality indicators and a range of related instruments by which such assessments might in future be conducted. Europe-wide, we were able to implement the instruments in 10 different languages, applying 26 indicators. The centres were diverse in level of care, setting and structure and operated within different cultures and health systems. Implementation was readily acceptable and all centres were able to collect data quickly and efficiently in the nine domains deemed important for quality evaluation [3, 8]. Across centres, findings were comparable. Our study showed common trends in practice, and what was majority practice, and could therefore guide the setting of benchmarks against which quality might be judged. At the same time it uncovered deficits in individual centres, indicating need for more systematic use of diagnostic diaries and disability and quality of life instruments, and revealing the restrictions on opportunities for follow-up visits. In these ways our study confirmed that the indicators could be regarded as a guide to improve quality, were fit for purpose and had utility.

In some centres, the findings reflected local system difficulties. The structures of the centres were different: there were university hospitals and both public and private clinics with varied financial resources and differently structured health care systems. Many centres had no control over equal access. We saw that some centres had problems with follow-up: many specialised centres and universities were operating in one-touch systems, as a rule seeing their patients only once before sending them back to peripheral doctors with advice. These centres had no possibility to control the success of treatment; worse, from the point of view of audit and quality maintenance, they might never know whether their recommendations led to success or failure. Such systems do not inevitably lead to suboptimal care, but they certainly do not promote quality of care. Restrictions imposed by operating environments and resource limitations often lay behind absence of referral pathways and lack of access to some disciplines of care such as psychological therapy. The development of benchmarks based in part on practice in other centres may be an evidence-based means by which these restrictions can be lifted, if this requires action at political level.

Other deficits appeared to be within the control of centres. Diagnostic diaries, demonstrated to be very valuable both in guiding diagnosis and in setting treatment plans [19], were not universally available. Failure to have and make use of instruments for disability assessment — although disability is a major consequence of headache disorders and a range of well-validated disability instruments exists [1, 9, 12, 17, 21] — appears to be both necessary and easy to remedy. The same may apply to quality-of-life assessment, a problem in almost all centres, although this is more relevant to outcome assessment during follow up than to the formulation of treatment plans at time of presentation. The mindset of most of the centres appeared to focus on treatment of patients’ symptoms.

These findings — demonstrating to centres where standards are not met and improvements are needed — are both a call for and a guide to action. We saw differences between newer and older centres, the former still working on the implementation of standards. There were different levels of knowledge and experience among HCPs: some centres were run only by permanent headache-experienced HCPs, others — especially those with training roles — made use of HCPs with less experience and relatively high turnover. In some centres HCPs were not familiar with the overall structure of their service, or aware of the existence of special protocols or processes. Here our findings are a guide for extra teaching in these centres.

More patients than HCPs considered that the service environment was clean and comfortable. There was generally more dissatisfaction with waiting times and times per visit among HCPs than patients. Overall satisfaction with their management was expressed by most patients, emphasising the rewards that a good headache service can bestow on its patients (and, more broadly, all society, which bears a substantial part of the burden of headache [15]). These findings raise the question: What are the expectations of patients? Neither waiting time nor time allocated to patients’ visits appeared to influence patient satisfaction. A thorough evaluation of service quality may need to look more closely into the reasons for satisfaction or dissatisfaction of patients: while the original formulation of the quality indicators took patients’ expressed preferences into account [7], there may be intercultural differences. Most patients suffering from headache who receive timely and good-quality health care can expect this care to be effective, restoring quality of life [12], but good quality in health care has not been the automatic result of the marked changes in scope, character and content of headache practice and care that have occurred during the last years. Quality of care has not been much subject to social awareness or interest: our study, in the context of the collaborative EHF/LTB project of which it is part, is a step towards bringing headache service quality centre-stage. This takes importance from the fact that there has been no similar initiative preceding it.

The strengths of our study are its international scope, inclusion of many diverse centres within different cultures and interrogation of all three interested groups: HCPs, other health-care workers and almost 700 patients. The principal limitation was that we did not collect any information about the quality of the treatment itself. In a full quality evaluation, external expert reviews would check additional quality indicators relating to treatment in each centre by retrospective examination of the records [8]. However, we did not have the resources to undertake this part.

It is a matter for further studies to set benchmarks. A key pertinent question is: does majority practice correctly indicate what the benchmark should be, or might acceptance of majority practice in quality indicators crystallise suboptimal practice? In relation to this, future studies might question whether availabilty of referral pathways to psychological therapies and use of quality-of-life assessment are essential to quality; neither is supported by universal practice or evidence of cost-effectiveness. A second key pertinent issue is whether the same benchmarks are appropriate at all levels and in all settings, on the basis that quality is an invariable construct. Also in future studies, the following might be subjects of especial scrutiny:

- Do waiting-list times for appointments actually reflect urgency of need?
- Do treatment plans follow evidence-based guidelines according to diagnosis?
- Are patients not over-investigated (special investigations of concern include MRI, CT, EEG, Doppler, evoked potentials, skull and neck xrays)?
- Are patients not over-treated (over-treatment may mean excessive use of drugs likely to induce medication overuse headache (MOH), overdosage with potentially harmful drugs such as ergotamine or steroids, use of prophylactics for infrequent headache, use of prophylactics for the wrong diagnosis, or use of non-evidence-based treatments that are unlikely to be effective and may jeopardize safety)?

## Conclusions

In conclusion, this Europe-wide study was the first study anywhere to evaluate headache care quality indicators across a culturally diverse multinational range of settings. It showed broad commonalities while uncovering deficits that might be remedied, and demonstrated that the indicators were fit for purpose and could be a guide to quality improvement, at least in specialist care. It highlighted lack of systematic use of diagnostic diaries and disability and quality-of-life assessment instruments, and restricted opportunities for follow-up visits. It showed what was majority practice and, whether based on this or not, can guide the setting of benchmarks against which quality may be judged.

The next step is to take the process into non-specialist care, since this is where most headache patients are and should be treated [12, 22]. The finally-agreed quality indicators should not themselves be varied when taken into primary care, even though the benchmarks might be different.

## Additional files

Additional file 1: SQE implementation questionnaire doctor. (PDF 181 kb)
Additional file 2: SQE implementation questionnaire manager. (PDF 255 kb)
Additional file 3: SQE implementation questionnaire nurse or other HCP. (PDF 251 kb)
Additional file 4: SQE implementation questionnaire patient. (PDF 181 kb)
Additional file 5: SQE implementation questionnaire secretary. (PDF 162 kb)
Additional file 6: Problems highlighted at centres and explanatory commentaries. (DOCX 34 kb)

## Abbreviations

AV: Alexander vein
EHF: European headache federation
HALT: Headache-attributed lost time questionnaire
HCPs: Health-care providers
HURT: Headache under-response to treatment questionnaire
ICHD: International classification of headache disorders
LTB: Lifting the burden
MOH: Medication-overuse headache
SD: Standard deviation
TTH: Tension type headache
U: University
WHO: World health organization
WHOQoL: The world health organization quality of life questionnaire
