# Supplementary material for: Headache service quality: evaluation of quality indicators in 14 specialist-care centres
Source: J Headache Pain. 2016 Dec 8;17(1):111. doi: 10.1186/s10194-016-0707-9 (PMC5145818; doi:10.1186/s10194-016-0707-9)
Supplement: Additional file 3: — SQE implementation questionnaire nurse or other HCP. (PDF 251 kb) [file 10194_2016_707_MOESM3_ESM.pdf]

Lifting The Burden
in Official Relations with the World Health Organization
The Global Campaign against Headache

UNIVERSITY OF OXFORD

Add logo of local collaborator

Research Project: Evaluation of headache service quality

[list the local collaborator(s)]

Other health-care providers' questionnaire

Under the auspices of the Global Campaign against Headache, headache centres in Europe and the United States, and the University of Oxford in the UK, are collaborating to define and measure *quality* of headache care. This is a step towards making improvements in headache care at local, national and international levels.

A multi-dimensional definition of quality has been agreed, along with a set of quality indicators. This study is an evaluation of the quality indicators implemented in specialist headache centres. Health-care providers, patients and administrative staff are asked to complete short questionnaires, and a review of randomly selected patients' records is conducted.

If the study is successful, it will be followed by studies in other settings, including primary care. Ultimately, the quality indicators, once validated, will be used to improve headache services.

There are five short questionnaires. This one of 13 questions is aimed at health-care providers other than doctors. Please complete it by ticking the relevant box for each question. Once you have done so, please return it to [local collaborator].

First please indicate whether you are:

Nurse O Physiotherapist O Psychologist O Other O

and the date of completion: ____/____/20___

	Office use only
**1. Are you satisfied that sufficient time is allocated to each patient's visit to enable a good management?**<br>O yes<br>O no	**B2c**
**2. Does an access route to psychological therapies exist in your headache service?**<br>(these may be provided within your own service or by direct referral to another service)<br>O yes<br>O no<br>O don't know	**B4b**
**3. Is an instrument for disability assessment available in your headache service?**<br>O yes<br>O no<br>O don't know	**B5b**
**4. Does your headache service allow follow-up of every patient who needs it?**<br>O yes<br>O no<br>O don't know<br>**5. Is a follow up diary or calendar available in your headache service?**<br>O yes<br>O no	**B6a**<br><br><br><br>**B6d**
**6. Are information leaflets for headache patients available in your headache service?**<br>O yes<br>O no<br>O don't know	**D1**
**7. Are you satisfied with the cleanliness and comfort of the environment in your headache service?**<br>O yes<br>O no	**E1b**

**8. Do you in general think patients' waiting times in the clinic are acceptable?**<br>O yes<br>O no	**E3b**
**9. Is your headache service equally accessible for all patients who need it?**<br>(if access to the service depends on ability to pay or another restriction unrelated to clinical need, answer "no")<br>O yes<br>O no<br>O don't know	**G4**
**10. Is an outcome measure available in your headache service that is based on self-reported symptom burden**<br>O yes<br>O no<br>O don't know	**H1b**
**11. Is an outcome measure available in your headache service that is based on self-reported disability burden?**<br>O yes<br>O no<br>O don't know	**H2b**
**12. Is an outcome measure available in your headache service that is based on self-reported quality of life?**<br>O yes<br>O no<br>O don't know	**H3b**
**13. Does a protocol (rules and written procedures) for reporting serious adverse events exist in your headache service?**<br>O yes<br>O no<br>O don't know	**I2b**

Please add further comments, if any, below:

Thank you for completing the questionnaire. Please return it to [local collaborator]
