# Supplementary material for: Headache service quality: evaluation of quality indicators in 14 specialist-care centres
Source: J Headache Pain. 2016 Dec 8;17(1):111. doi: 10.1186/s10194-016-0707-9 (PMC5145818; doi:10.1186/s10194-016-0707-9)
Supplement: Additional file 5: — SQE implementation questionnaire secretary. (PDF 162 kb) [file 10194_2016_707_MOESM5_ESM.pdf]

Lifting The Burden
in Official Relations
with the World Health Organization
The Global Campaign against Headache

UNIVERSITY OF OXFORD

Add logo of local collaborator

Research Project:
Evaluation of headache service quality

[list the local collaborator(s)]

Secretary/appointments administrator's questionnaire

Under the auspices of the Global Campaign against Headache, headache centres in Europe and the United States, and the University of Oxford in the UK, are collaborating to define and measure *quality* of headache care. This is a step towards making improvements in headache care at local, national and international levels.

A multi-dimensional definition of quality has been agreed, along with a set of quality indicators. This study is an evaluation of the quality indicators implemented in specialist headache centres. Health-care providers, patients and administrative staff are asked to complete short questionnaires, and a review of randomly selected patients' records is conducted.

If the study is successful, it will be followed by studies in other settings, including primary care. Ultimately, the quality indicators, once validated, will be used to improve headache services.

There are five short questionnaires. This one of only 4 questions is aimed at whoever is responsible for making or administering appointments. Please complete it by ticking the relevant box for each question. Once you have done so, please return it to [local collaborator].

First please indicate whether you are:

Secretary O Administrator O

and the date of completion: ____/____/20___

	Office use only
**1. Does a formal triage system exist in your headache service?**<br>(this means any system during the first telephone contact, or on receipt of a referral letter, that identifies patients' particular needs and reacts accordingly)<br>O yes<br>O no	**B1a**
**2. Is your triage system designed to pick out potentially urgent cases for early appointments?**<br>(for example, patients with cluster headache)<br>O yes<br>O no	**B1a**
**3. Does a referral pathway exist from primary care to your headache service?**<br>O yes<br>O no	**C1b**
**4. Does this pathway permit, and respond to, urgent referral when needed?**<br>O yes<br>O no<br>O not applicable	**C2b**

Please add further comments, if any, below:

Thank you for completing the questionnaire. Please return it to [local collaborator]
