# Supplementary material for: Headache service quality: evaluation of quality indicators in 14 specialist-care centres
Source: J Headache Pain. 2016 Dec 8;17(1):111. doi: 10.1186/s10194-016-0707-9 (PMC5145818; doi:10.1186/s10194-016-0707-9)
Supplement: Additional file 6: — Problems highlighted at centres and explanatory commentaries. (DOCX 34 kb) [file 10194_2016_707_MOESM6_ESM.docx]

## Supplemental data. Problems highlighted at centres and explanatory commentaries

| **Headache centre** | **Highlighted problem** | **Explanatory commentary** |
| --- | --- | --- |
| Austria-Linz | No protocol to limit wastage (G1): | *“There is no need for such a protocol.”* |
|  | No record of input costs (G2): | *“Cost efficiency should not be considered as a main aspect when treating patients in the best way possible; however there is a necessity for being aware of incidental costs.”* |
|  | No protocol for reporting serious adverse events (I1): | *“Serious adverse events are of course reported in patients’ electronic health records, but not according to a specific protocol.”* |
| Belgium-Ghent | No formal triage system (B1a): | *“A formal triage system does not exist but secretaries know that urgent cases, such as cluster headache patients, have to be seen immediately. If serious secondary headaches are suspected, patients are referred to the emergency room.”* |
|  | No access route to psychological therapies (B4): | *“Multidisciplinary approach is difficult to realise in Ghent because of previous political decisions made on the basis of a report by an expert group on chronic pain. This group, with no representative from neurology/headache departments, suggested that chronic headache patients should be referred to one of the 35 multidisciplinary pain centres in Belgium subsidized for this purpose. The problem of this system is that these clinics mainly employ anaesthesiologists without specific training in headache care. The neurology department in Ghent is not able to support multidisciplinary headache care financially and patients must pay for treatment themselves. In order to keep the costs affordable a formal collaboration cannot be installed but patients can be referred to psychologists or physiotherapists.”* |
|  | No instrument for disability assessment (B5): | *“We do not routinely do disability assessments (except for example for clinical studies), because residents are under much time pressure and it would be just on more thing to do.”* |
|  | No information leaflets are available (D1a): | *”Dutch, French and English versions of Lifting The Burden booklets on migraine, cluster headache, tension-type headache and chronic daily headache are available, but this is apparently insufficiently known by junior residents.”* |
|  | HCPs were not satisfied with waiting time (E3c): | *“There is especially a lot of pressure on HCPs for (semi-) urgent referrals.”* |
|  | No protocol to limit wastage (G1): | *“There are no such protocols in the entire neurology department.”* |
|  | No record of input costs (G2): | *“It is not possible to assess cost efficiency as the headache service belongs to the pool of the neurology department.”* |
|  | No evaluation of quality of life (H1+2+3): | *“Owing to time constraints it is not possible to assess outcome measures/QoL routinely.”* |
|  | No protocol for reporting serious adverse events (I2): *“* | *“A formal system for reporting serious adverse events exists but is rarely used for outpatients.”* |
| Denmark-Copenhagen | Patients are not always reassured by doctor (D2): | *“Most patients are referred as refractory or difficult to treat and in general they have a long headache history and very high expectations of treatment. The time constraint for the initial consultation may be a problem for sufficiently reassuring but we have the opportunity for several follow-up visits where the need for further reassurance can be met.”* |
|  | HCPs were not satisfied with service environment (E1a): | *“The Headache Centre is temporarily located in old and insufficient facilities but a new building specifically designed for it is under construction.”* |
|  | HCPs were not satisfied with waiting time (E3c): | *“Our waiting time can be up to 2 years because of excessive need for and interest in specialized headache treatment in Denmark. The long-lasting suffering and disability are frustrating for the HCPs, as patients may have had better outcomes if they had had the chance for earlier optimized treatment.”* |
| Estonia-Tartu | No formal triage system (B1a): | *“There is no such system.”* |
|  | Not all HCPs are satisfied with time spent per visit (B2c): | *“Highly individual evaluation in any case.”* |
|  | No access route to psychological or physiological therapies (B4): | *“Firstly, physio can be used in only selected cases with patients having to pay for themselves. Secondly, there is no psychologist attached to the the department. However, until now, a headache/pain-specialist psychologist working part-time at another clinic of the institution (rehabilitation) is available for headache patients. There are also agreements with two private clinics for this service, not covered by national insurance. These are the main issues the clinic is working on at the moment. In these therapy fields there is a lack of awareness and acceptance of headache as such an enormous problem, and insufficient financial support, which make us unable to hire the staff needed.”* |
|  | No intrument for disability assessments (B5): | *“There is not.”* |
|  | HCPs were not satisfied with service environment (E1a): | *“The space allocated for our service is ridiculously small and uncomfortable. HCPs feel this.”* |
| Georgia-Tbilisi | Duration and frequency of complaint/symptoms not always recorded (A1a+b): | *“The headache clinic has been recently established and corresponds with EHF/LTB level 2 (non-university setting, patients are seen by neurologists with some training in the field). This SQE study revealed deficiencies of this setting that need to be improved.”* |
|  | Not always working diagnosis at first visit recorded (A3): |  |
|  | No access route to psychological therapies (B4): | *“Psychological therapy is neither well-developed nor cost-effective in Georgia.”* |
|  | HCPs were not satisfied with waiting time (E3c): | *“This problem refers to patients who are self-referred without appointments.“* |
|  | No protocol to limit wastage (G1): | *“Management of the service needs to be be optimized.”* |
|  | No evaluation of quality of life (H1+2+3): | *“Outcomes were assessed according to symptom burden (frequency and intensity of headache). This SQE study revealed that in addition we should pay attention to quality of life.”* |
|  | Additional remarks: | *“Weak triage system: The referral of patients from level 1 to level 2 is difficult because the network within the health-care system does not work properly.”*  *“Low socio-economic level: The deficits in socio-economic affairs affect the quality of the headache service available in Georgia. Headache services on a high level are currently developing, and more time, experience and resources are needed in order to reach this aim.”* |
| Germany-Munich | Temporal profile is less recorded than in other centres (A1a+b): | *“This problem is not seen as a general drawback but rather an individual or cultural tendency of anamnesis. Some doctors prefer more general information about the patient’s temporal profile, such as stages of life, instead of exact dates in order to arrive at their diagnoses. Exceptions are new daily persistent headache and the anamnesis of secondary headaches, where concrete dates are relevant information.”* |
|  | No instrument for disability assessment (B5): | *“The MIDAS scale is used in the majority of the patients.”* |
|  | Not every patient received follow-up who needed it (B6a): | *“The centre sees its duty as informing patients about their headaches which includes diagnosis, acute and prophylactic therapy and discussing their expectations. Subsequent to this consultation, patients are expected to make appointments with a local headache specialist near their homes. The specialist supervises the therapy but if necessary the patient can be referred to the clinic a second time. Therefore the clinic is able to treat more patients with urgent needs for treatment.”* |
|  | No referral pathway for urgent cases (C2): | *“All urgent cases can be seen in the emergency room where a neurologist is available 24 hours a day, 7 days a week.”* |
|  | No protocol to limit wastage (G1): | *No comment.* |
|  | Not controlling for cost efficiency (G2): | *“In an academic environment and in order to give patients the best accessible treatment, cost efficiency should not be considered as a main aspect although the necessity for controlling costs in the private sector is understood.”* |
|  | No protocol for reporting serious adverse events (I1): | *“The idea of introducing a standard protocol for side effects is being reconsidered.”* |
| Germany-Unna | No instrument for disability assessment (B5), no protocol to limit wastage (G1), no control for cost efficiency (G2), no QOL instruments for outcome (H1+2+3), no protocol for reporting serious adverse events (I1): | *“The headache clinic started work two years ago. We did not realize the deficits mentioned above. The problems have became clear during the study.”* |
| Italy-Rome | Not all HCPs were aware of the formal triage system (B1a): | *“Not all patients call the Headache Centre directly to make an appointment. A relevant percentage come via RECUP (Regional Centre of Unique Prenotation [*[*http://www.regione.lazio.it/rl_sanita/?vw=contenutidettaglio&id=43*](http://www.regione.lazio.it/rl_sanita/?vw=contenutidettaglio&id=43)*]), the regional public health prenotation systems, who are not informed about the formal triage system.”* |
|  | No sufficient access route to psychological therapies (B4): | *“In the past years psychological therapies were included in reasearch protocols. Now a public access via RECUP is available for a psychological support protocol in medication-overuse headache patients.”* |
|  | No sufficient policy to ensure equal access (G3): | *“There are three different ways to access the Headache Centre: through RECUP, through Sant’Andrea CUP and urgently through both the Emergency Department of our hospital and general practitioners with direct online requests.”* |
|  | Not all HCPs were aware of the possibility to evaluate quality of life (H1+2+3): | *“Questionnaires on quality of life have always been included in medical and psychological protocols but, given the time assigned for each first and control visit, there has not always been the chance to inform or hand the questionnaire out.”* |
| Norway-Trondheim | No diagnostic diaries available (A6a): | *“We use non-diagnostic diaries that focus on frequency and intensity of headache, and on the frequency and type of acute medication. Diagnosis is based on this in combination with patients' histories.”* |
|  | Lack of access route to psychologist (B4): | *“There is a deficit in monetary funding of the headache service centres and their work. Therefore an expensive psychological programme cannot be established yet.”* |
|  | Not every patient in need of follow-up receives it (B6a): | *“There is only a limited number of physicians who face an enormous number of patients from within and outside the hospital. In order to manage this problem the priority is to treat urgent cases first. If patients do not receive follow-up although they need it, it is the consequence of this strategy.”* |
|  | HCPs were not satisfied with waiting times (E3c): | *“HCPs are not satisfied as long as some patients are not.”* |
|  | Some HCPs report no policy to ensure equal access (G3): | *“There is a policy to ensure equal access.”* |
|  | Most HCPs report no protocol for reporting serious adverse events (I1): | *“There is a protocol for reporting serious adverse events, which is valid in the whole Norwegian healthcare system.”* |
| Portugal-Lisbon | HCPs not aware of diagnostic diaries (A6a), access route to psychological therapies (B4), instruments for disability (B5), referral pathways (C1) or information leaflets (D1a): | *“In Lisbon, referral pathways, priority policies, access route to psychological therapies, diagnostic diaries, instruments of disability and information leaflets exist and are available but not all HCPs are aware of them. This is related to the geographic dispersion between HCPs in the headache centre, as it works as a functional unit within the hospital but has yet no physical departmentalization. As a consequence, doctors, nurses and therapists are physically separate although clinical information is synchronized electronically. This study has identified the need to make these instruments available at all sites and improve team coordination.”* |
|  | No protocol to limit wastage (G1) and no record of input costs (G2): | *“The headache centre has no independent management or budget allocation, so it is not yet possible to evaluate any financial aspects of its service. The centre’s expected growth may allow independent management in the future. ”* |
|  | Some HCPs report no policy to ensure equal access (G3): | *“Hospital da Luz is a private hospital. All patients have equal access irrespective of their health-care plan or health-care system and can also access care directly, supporting their costs themselves. Access is not limited by any clinical or regional aspect; self-referral is also possible.”* |
|  | No protocol for reporting serious adverse events (I1): | *“This important aspect was identified by the study and will be subject to protocol review in the near future.”* |
| Russia-Moscow, Alexander Vein | Often frequency or duration of symptoms are not recorded (A1a+b): | *“Medical records are not based on a formal structure. Some doctors tend to write down very brief patient records.”* |
|  | No instrument for disability assessment (B5): | *“There is no reimbursement from insurance companies, so no formal questionnaries or asessments are required. We always ask the patient’s opinion about his/her disability level to optimize treatment.”* |
|  | No referral pathway and no referral pathway for urgent cases (C1+2): | *“No formal referral because of the lack of reimbursements. Physicians may recommend the patient to come to us, and many patients self-refer because the Clinic is well-known across Russia. There is almost always an opportunity to see an urgent patient (mostly cluster headache) on the same day, but no formal guarantee.”* |
|  | Some HCPs were not satisfied with service environment (E1a): | *“We moved to a new location which at the time of the study had problems with climate control. This may have caused some HCPs to be dissatisfied.”* |
|  | No protocol to limit wastage (G1): | *“This flaw became clear to us only during this study.”* |
|  | No policy to ensure equal access (G3): | *“This is due to the lack of insurance reimbursement. Most patients pay out-of-pocket. However we keep prices relatively low to make access easier.”* |
|  | No protocol for reporting serious adverse events (I1): | *“This necessity is now being considered. However there is no supervising body that requires this information.”* |
| Russia-Moscow, University | Diagnosis is not routinely reviewed at follow-up (A5) and triage system does not expedite appointments of urgent cases (B1b): | *“As chronic headache treatment is a relatively new focus in Russia its physicians are not yet provided with the most recent knowledge of efficient therapeutic options.”* |
|  | No control over cost efficiency (G2): | *“We are controlling cost efficiency, but although a record exists of input costs the clinic states that its control of cost efficiency is deficient.”* |
|  | No policy to ensure equal access (G3): | *“The complex multidisciplinary treatment for chronic headache or medication-overuse headache is too expensive as it is not covered by insurance and patients have to pay themselves.”* |
| Serbia-Belgrade | HCPs are dissatisfied with the actual time per visit (B2c): | *No comment.* |
|  | No access route to psychological treatments (B4): | *“Psychologists and physiotherapists are formally available for patients who need their services, but headache patients do not have a priority, and are often left without it.”* |
|  | No instrument for disability assesment (B5): | *No comment.* |
|  | No information leaflets (D1a): | *No comment.* |
|  | Some HCPs were not satisfied with service environment (E1a): | *No comment.* |
|  | Long waiting times (E3a) and some patients and HCPs were dissatisfied with waiting times (E3c): | *“Besides long waiting times and waiting lists we find this to be a serious problem which arises from a low awareness and marginalized position of headache care among neurologist in Serbia, just as it is in the general society.”* |
|  | No record of input costs (table 4, G2): | *No comment.* |
| Turkey-Istanbul | Some HCPs and some patients were not satisfied with actual time per visit (B2b+c): | *No comment* |
|  | Lack of psychological route access (B4): | *“When the questionnaire was filled we had two psychologists who were working voluntarily. One left and since then they could not perform all tests regularly.”* |
|  | No information leaflets available (D1a): | *“We have headache diary for every patient and a leaflet including the phone number of our HCP to access our institute immediately.”* |
|  | Long waiting times (E3a) and dissatisfaction with waiting time (E3b+c): | *“Because of the study we could see dissatisfaction with waiting times among patients and we are going to work on this drawback.”* |
|  | No record of input costs (G2): | *“The interventional treatments for chronic headache may not be covered by insurance and patients sometimes have to pay the expenses themselves.”* |

HCPs: health-care providers.
